# Supplementary material for: MetaRibo-Seq measures translation in microbiomes
Source: Nat Commun. 2020 Jun 29;11:3268. doi: 10.1038/s41467-020-17081-z (PMC7324362; doi:10.1038/s41467-020-17081-z)
Supplement: Supplementary file 10 — Supplementary Data 7 [file 41467_2020_17081_MOESM10_ESM.zip › File2/Confidence_VeryHigh_Taxonomy/161993_out.krona.html]

Javascript must be enabled to view this page.

members
magnitude
magnitudeUnassigned
count
unassigned
taxon
rank

161993\_out

5

superkingdom
2
5

1239
5
phylum

5
186801
class

order
5
186802

5
1898207

SRS018836\_contig\_number\_contig-100\_36466.85012SRS022609\_contig\_number\_contig-100\_18696.18696SRS045645\_contig\_number\_contig-100\_2208.180825SRS054352\_contig\_number\_24528SRS104693\_contig\_number\_3450
species
